# Effectiveness of Virtual Reality Systems to Improve the Activities of Daily Life in Older People

**DOI:** 10.3390/ijerph17176283

**Published:** 2020-08-28

**Authors:** Ana-Isabel Corregidor-Sánchez, Antonio Segura-Fragoso, Juan-José Criado-Álvarez, Marta Rodríguez-Hernández, Alicia Mohedano-Moriano, Begoña Polonio-López

**Affiliations:** 1 Faculty of Sciences Health, University of Castilla la Mancha, 45600 Talavera de la Reina, Spain; Antonio.Segura@uclm.es (A.S.-F.); jjcriado@sescam.jccm.es (J.-J.C.-Á.); Marta.RHernandez@uclm.es (M.R.-H.); Alicia.Mohedano@uclm.es (A.M.-M.); Begona.polonio@uclm.es (B.P.-L.); 2 Institute of Sciences Health, 45006 Castilla la Mancha, Spain

**Keywords:** virtual reality, functional autonomy, rehabilitation, exergame

## Abstract

(1) This review aims to evaluate the effectiveness of treatments with virtual reality systems (VRSs) on the functional autonomy of older adults versus conventional treatment. (3) Methods: Systematic review and meta-analysis. An electronic data search was carried out, following the PRISMA statement, up to February 2020. We combined results from clinical trials using VRSs for the improvement of basic and instrumental activities of daily living. The guidelines of the Cochrane Handbook for Systematic Reviews of Interventions were followed for calculations and risk of bias. The Grading of Recommendations Assessment, Development and Evaluation (GRADE) was used to assess the quality of evidence. (4) Results: The final analysis included 23 studies with a population of 1595 participants. A moderate, but clinically significant, effect was found for basic activities of daily living (BADLs), (Standard Medium Deviation, SMD 0.61; 95% CI: −0.15–1.37; *P* < 0.001). A small effect was found for instrumental ADLs (Instrumental Activities of daily living, IADLs) (SMD −0.34; 95% CI: −0.82–0.15; *P* < 0.001). Functional ambulation was the BADL which improved the most (SMD −0.63; 95% CI: −0.86, −0.40; *P* < 0.001). (5) Conclusion: The use of VRSs is an innovative and feasible technique to support and improve the functional autonomy of community-dwelling older adults. Due to the very low quality of the evidence for our main outcomes, the effects of a VRS on the BADLs and IADLs are uncertain. Clinical trials of a higher methodological quality are necessary to increase the level of knowledge of its actual effectiveness.

## 1. Introduction

The morphological and functional changes associated with aging can have a negative impact on the performance of activities of daily living (ADLs). It has been estimated that 20–25% of community-dwelling people over 75 may experience limitations in their capacity to perform ADLs [1,2]. This dependency is a predictor of frailty and institutionalization [3,4]. In a recent study, the American Occupational Therapy Association (AOTA) supported the need to build a friendly environment and to implement prevention programs which include physical, social and leisure activities for older persons with a high and stable intrinsic capacity [3].

In the last decade, there has been a growing interest in the use of virtual reality systems (VRSs) to implement programs for supporting the functional ability of older adults through physical exercise [5,6,7], cognitive [8,9] and social interventions [10]. VRSs allow for the re-creation and control of virtual everyday environments, and for the planning of safe, ecological and motivating treatment activities. Unlike the real world, the virtual world can be adapted and adjusted to the capacities and needs of each person, and thus it provides great flexibility of experiences and virtual tasks, which include the sequences of functional movements necessary to achieve autonomy in real-life activity. The analysis and control of all the activity’s virtual elements by the occupational therapist enable virtual achievements which could not be attained in real life, due to either disability or to environment restrictions. These achievements increase motivation, commitment and adherence to the rehabilitation process.

The possibility of generating exercises that can be repeated over time, selecting the frequency and intensity, enables compliance with the principles of motor learning and neuroplasticity, as well as the generalization of this learning [11]. A new performance context is therefore created, with which the person identifies and gets involved. Thus, they manage to carry out actions which are not always possible in their original setting and which can help to improve their occupational performance [12]. These characteristics make virtual reality-based technology an appropriate rehabilitation technique for different people.

Various reviews have been published in recent years about the effectiveness of VRSs for older adults. These have shown positive effects on physical functioning [6,13,14] and cognitive functioning [15,16]. However, no previous review has analyzed the effects of VRSs on autonomy in the performance of activities of daily living in older adults. In addition, many of the previous reviews have focused on older populations with neurological disorders, excluding older adults without disabilities. Thus, the preventive potential of VRSs is still not known.

The aim of this systematic review and meta-analysis is to critically evaluate the effectiveness of VRSs for improving the functional autonomy of older populations and for preventing disability. These findings can be useful for future research and intervention projects aiming at preventing disability and promoting the occupational performance of older adults.

## 2. Materials and Methods

The Preferred Reporting Items for Systematic Reviews and Meta-Analyses (PRISMA) statement was employed to carry out this review [17]. The Grading of Recommendations Assessment, Development and Evaluation was also used [18].

### 2.1. Search

Six databases were searched electronically (Web of Science, OT Seeker, Guideline National Clearing House, databases of the Spanish National Research Council (CSIC), Scopus and Cochrane), up to February 2020. No date, study design, language or age limits were set in order to increase the sensitivity of the search. The electronic search was completed with a hand search of the bibliographical references of the studies included. The search terms were developed by a methodology consultant from the Institute of Health Sciences (ICS, Spain). See Appendix A for key search terms.

The inclusion criteria were the following: (1) studies with older adults over 60 years of age who performed activities of daily living independently, (2) studies which used VRSs in order to improve the performance of ADLs [19], (3) studies whose outcome measures included the assessment of ADLs, (4) studies considered as peer-reviewed scientific literature (Level I, Level II or Level III) [20] and (5) studies with a simple majority in the 25 items of the CONSORT checklist [21].

The studies excluded were those with participants under the age of 60 with neurological or osteoarticular disorders which severely affected functional independence, studies which did not use VRSs directly and studies which did not report on changes in occupational performance.

### 2.2. Review, Selection and Data Extraction

Two independent reviewers selected the studies. Discrepancies were resolved by a third reviewer. The data extraction protocol was based on the PRISMA items [17] and the Cochrane Handbook for Systematic Reviews of Interventions. The form included information regarding methodological characteristics, participants, healthcare resource, intervention, results on functional ability and conclusions. Study authors were contacted to retrieve unreported data.

### 2.3. Summary Measures and Statistical Analysis

Various outcome measures were used. Objective measures were included which evaluated ADLs in an overall way (Barthel Index [22], Functional Independence Measure (FIM) [23], Late Life Function and Disability Instrument (LLF&DI) [24] and the Instrumental Activities of Daily Living Scale [25]). Quantitative assessment tools were also included which assessed specific ADLs, such as ambulation or transfers (Functional Gait Assessment (FGA) [26], Timed Up and Go (TUG) [27], Four Step Square Test (FSST) [28], 6-min walk test (6MWT) [29], 8-foot up and go [30] and Five Sit to Stand [31]). The effect magnitude was measured using Hedges’s adjusted g standardized mean difference (SMD), with its confidence interval at 95% (95% CI). The total effect size, weighted by the sample size of the studies, was calculated using the inverse variance method and a random effects model. Its 95% CI and its statistical significance were calculated using the *Z*-test. The effect size was interpreted using Cohen’s criteria for pooled estimates [32]; SMD > 0.20, small; SMD > 0.50, medium; and SMD > 0.8, large effect.

### 2.4. Assessment of Level of Evidence of the Set of Studies

The GRADE system [18] was used, considering eight factors to reduce or increase the level of evidence. The factors for downgrading the level were (1) risk of bias; (2) inconsistency; (3) indirect evidence; (4) imprecision by the calculation of the optimal information size (OIS); (5) publication bias. The factors considered for upgrading the level of evidence were (1) large effect size (SMD ˃ 0.8); (2) dose–response effect; (3) control for confounding factors in the individual studies.

### 2.5. Risk of Bias Assessment in Individual Studies

The risk of bias of each article was assessed independently by two reviewers using the items included in Review Manager (RevMan), version 5.3. (The Nordic Cochrane Centre, The Cochrane Collaboration: Copenhagen, Denmark, 2014). Based on five “risk of bias” items, we determined that studies at:

- a low risk of bias were those in which all items were assigned a low risk of bias;
- an unclear risk of bias were studies in which one or more items were found to be at an unclear risk of bias; and
- a high risk of bias were studies in which one or more items were found to be at a high risk of bias.

### 2.6. Heterogeneity

The I^2^ statistic was calculated, which was interpreted as absent (0), low (25), moderate (50) or high (75 or higher).

### 2.7. Publication Bias

Publication bias was assessed using a funnel plot created with RevMan, complemented with a DOI plot created with METAXL. Egger’s method, Begg’s test with Epidat 3.1 and the Luis Furuya-Kanamori (LFK) index were used. An LFK index ≤ 1 was considered as no asymmetry, ˃1 ≤2 as minor asymmetry and ˃2 as major asymmetry.

## 3. Results

**Figure 1:**
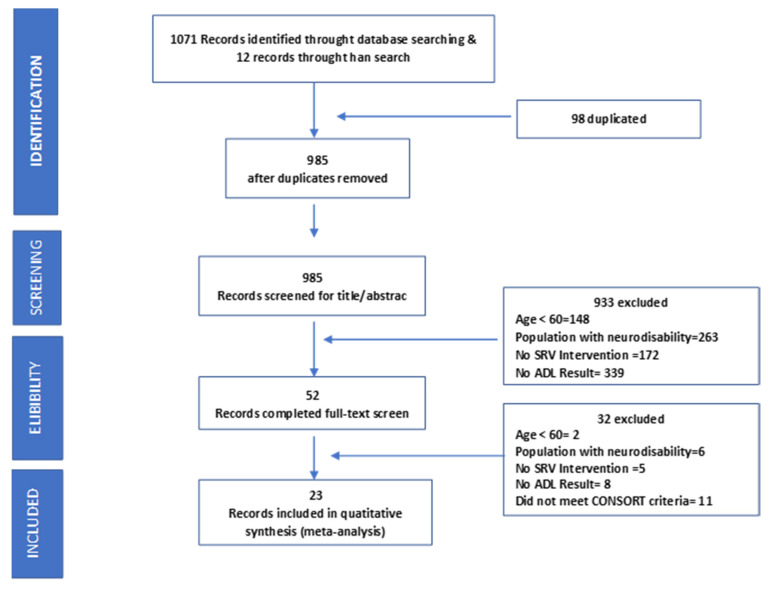
PRISMA flow diagram of search strategy and results.

In total, 1083 articles were identified. After removing duplicates, 985 records were screened, with 52 manuscripts selected for complete reading. The full text review included 23 studies applying VRSs to improve the functional ability of independently living older adults over 60 years of age. Figure 1 shows the flow chart of this selection. The total number of participants was 1595, with an age range of 60–96 years.

### 3.1. Characteristics of Included Studies

The included studies were published within the last nine years (2011–2020). That indicates that the use of VRSs is a very recent research area. The aim of these studies was to analyze the effect of VRSs on two areas of occupation and IAVDs; functional ambulation, the capacity to make transfers, cognitive function, physical condition and quality of life. Data about design, sample size, characteristics of the population, type of intervention and assessed outcomes are shown in Appendix B.

Five studies evaluated the overall impact of VRSs on BADLs [33,34,35,36,37] and three studies examined IADLs [38,39,40]. We also identified six studies which assessed the effectiveness of VRSs on the capacity to make transfers and twenty-one studies which reported on their effectiveness in improving functional ambulation.

The studies were randomized and quasi-randomized clinical trials. Eighteen studies were Level I (randomized controlled trials) and five studies were Level II (two groups, nonrandomized studies). The control groups received interventions consisting of physical exercise sessions (six studies) and health education (three studies). The remaining fourteen studies did not design any intervention for the control group. Only five studies carried out a follow-up after the intervention [38,39,41,42,43].

### 3.2. Intervention with VRS

The use of VRSs was the main intervention technique. Seven studies used VRSs specifically designed for rehabilitation [34,39,42,44,45,46,47]. The rest of the studies used virtual reality active video games (Nintendo^®^ Wii, XBox^®^, Sony^®^ PlayStation and Xavi Sport^®^). No study used head-mounted displays. Appendix C shows the devices and exergames used in each article. The duration of the interventions was from 1 to 24 weeks, with sessions between 20 and 50 min.

### 3.3. Effectiveness of VRSs on Activities of Daily Living

**Figure 2:**
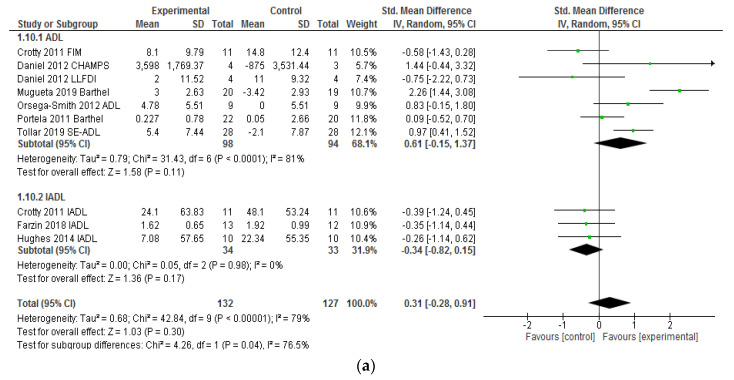

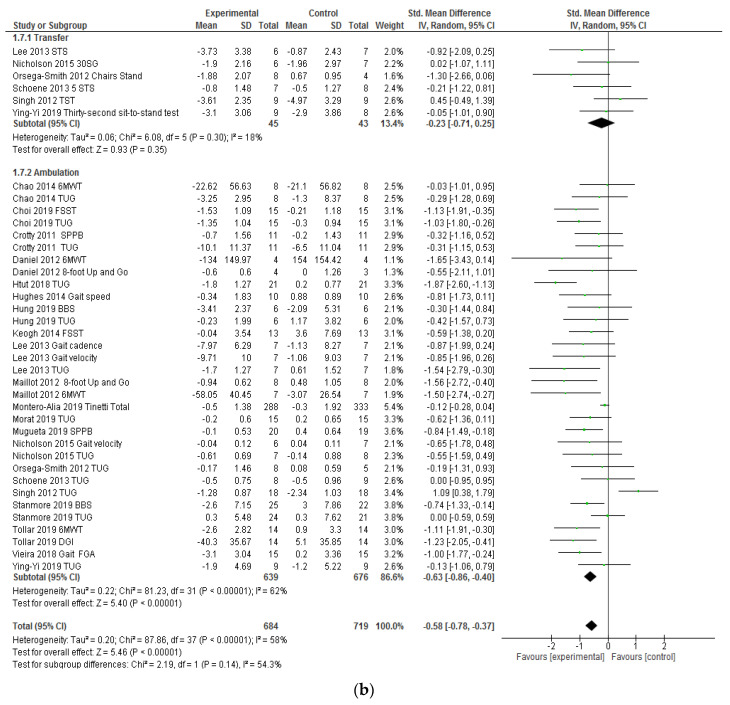
(**a**) Forest Plot illustrating effectiveness of VRSs on the activities of daily living (BADL and IADL). (**b**) Forest Plot illustrating effectiveness of VRSs on the transfer and functional ambulation.

Eight studies provided sufficient data to report on the effectiveness of VRSs in improving the performance of activities of daily living. As Figure 2a shows, the effect size of VRSs in improving the performance of BADLs is moderate but significant; the total SMD was 0.61 (95% CI: −0.15; 1.37). Four of the studies which assessed effectiveness on BADLs obtained a large effect (d > 0.8) [33,34,35,37] and two studies found a medium effect [33,38]. However, the effectiveness of VRSs was smaller for the three studies which assessed IADLs [38,39,40]. The total SMD was −0.34 (95% CI: −0.82; 0.15) and the effect found was small (Figure 2a). This indicates that the interventions with VRSs had a small non-significant effect (*p* = 0.3) for improving the performance of IADs compared to the control group.

### 3.4. Effectiveness of VRSs on Functional Ambulation and Transfers

Thirty-two outcomes from twenty-two studies provided sufficient data to analyze the effectiveness of VRS in the improvement of functional ambulation in older adults. The total SMD was −0.63 (95% CI: −0.86, −0.40, *p* = 0.001). This indicates that the interventions with VRSs had moderate, but significant effects (Figure 2a). The group of participants which received treatment sessions with a VRS increased their level of performance in ambulation compared to the control group that received traditional physical exercise.

Six outcomes of transfer ability from six studies were analyzed. The effect size of the treatment programs with VRSs on the ability to make transfers from sitting to standing and vice versa was small, with an SMD of −0.23 (95% CI: −0.71, −0.25; *P* < 0.001). Only two studies obtained a large effect [24,35], whereas the rest obtained a low effectiveness. Figure 2b shows the SMD for each study, as well as the total SMD.

### 3.5. Risk of Bias of the Individual Studies

**Figure 3:**
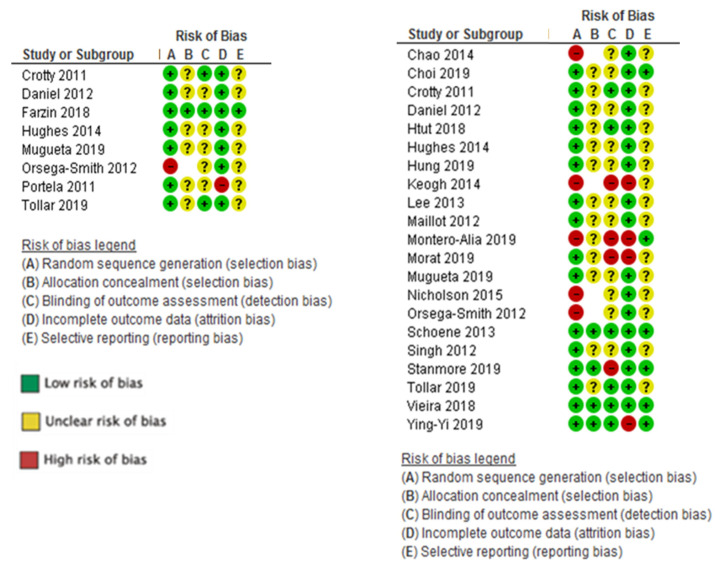
Risk of bias ADL studies, Risk of bias functional mobility studies.

Figure 3 shows the risk of bias assessment for each study. The risk of bias was moderate for the studies which addressed the effectiveness of VRSs in the performance of ADLs. Only one study did not do random sequence generation, and seven out of eight studies lacked clarification about the allocation concealment. Five studies (62%) did not blind outcome assessors and the incomplete outcome data bias was low.

The risk of bias for functional mobility and transfers was high (Figure 3). In functional ambulation, four studies had a high risk of bias for random sequence generation [35,48,49,50], none of the studies could blind participants, only six blinded outcome assessors [37,38,41,47,51,52] and only one had a low risk of bias for allocation concealment. On the other hand, loss to follow-up was significant in only one study (Keogh et al., 2014).

### 3.6. Heterogeneity

The studies which assessed the overall performance of ADLs (I^2^ = 79%) and transfers (I^2^ = 77%; chi-squared *p* = 0.0007) showed a high heterogeneity. In the studies which analyzed functional ambulation, heterogeneity was moderate, with I^2^ = 55% (chi-squared *p* = 0.004). The forest plots (Figure 2a,b) show a significant variability between the studies which favored the experimental intervention.

### 3.7. Publication Bias

**Figure 4:**
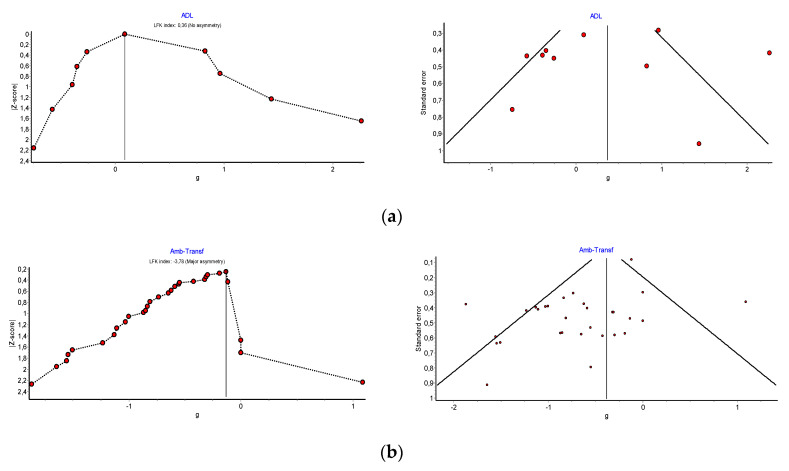
(**a**) Publication Bias: LFK Index and Doi Plot ADL Studies, (**b**) Publication Bias: LFK Index and Doi Plot Functional Mobility Studies.

The funnel plot and the DOI plot (Figure 4) show signs of publication bias. Both plots present asymmetry, mainly in those studies with fewer participants but a greater impact on functional performance. The LFK index showed minor asymmetry both for ambulation (LFK = 3.78) and for transfers (LFK = 0.36). The statistical significance of Begg’s test for ambulation was *p* = 0.0235 and that of Egger’s test was *p* = 0.0057. The statistical significance of Begg’s test for transfers was *p* = 0.13 and that of Egger’s test was *p* = 0.11.

### 3.8. GRADE Quality of Evidence

The quality of evidence was low to very low for studies which used VRSs to optimize ADL performance, and moderate to low for studies using VRSs to improve functional mobility. Appendix D specifies the reasons for decreasing the level in each of the study groups.

## 4. Discussion

This systematic review and meta-analysis examined the effectiveness and the quality of evidence of VRSs to support the functional autonomy of non-disabled older adults. Although there are still few studies that have analyzed the effectiveness of intervention programs based on virtual reality on older persons’ occupational performance, the results are encouraging. Seventy-three percent of studies showed significant improvement in the functional ability of this population group. However, the GRADE grading of recommendation was low to very low due to the risk of bias, inconsistency and imprecision of the analyzed data. Therefore, these studies should be treated with caution.

Additionally, clear signs of publication bias were found in studies which assessed functional mobility (LFK: −3.3). A detailed analysis showed that the studies with fewer participants had better results. This indicates the possibility that there might be unpublished studies which did not show significant effects of VRSs.

In most studies, the intervention with VRS was applied through commercial exergames or active video games; only seven studies used VRSs specifically designed for rehabilitation. This may be due to the accessibility and low cost of active game consoles. Moreover, commercial exergames offer a virtual re-creation of environments and challenges that are highly immersive, appealing and enjoyable for the user, thus increasing commitment to the task. In line with other reviews [53,54], our results suggest that the use of commercial virtual reality consoles, such as the Nintendo Wii^®^, PlayStation^®^ and Xbox Kinect^®^, can be beneficial and feasible in improving the functional autonomy of older persons.

We found that the use of VRSs is more effective and has greater evidence in the field of occupation corresponding to BADLs and ambulation than in the performance of IADLs. The reason for these results may be that IADLs are more complex activities, both in their treatment and their assessment, because they depend largely on the social and environmental context [55]. These factors are not addressed in the intervention programs based on VRSs. In this regard, recent reviews [56,57] conclude that improvement in physical performance does not always translate into improvement in IADLs. Nevertheless, we found a direct link between an increase in physical functional test scores (TUG, 6MWT) and the performance of basic activities. We assume, from these results, that intervention with VRSs could help older persons to live independently and safely in their own home and could facilitate productive aging.

This review and meta-analysis has several strengths. Its methodology did not set a time or language limit on the publications and made use of a wide range of databases in order to increase the sensitivity of the search. It also included articles of high methodological quality: 78% of the studies were Level I and the remaining 22% were Level II articles.

However, the articles included had a number of important methodological limitations: the sample size, the risk of bias and the post-intervention follow-up. Only two studies had more than 100 participants, and sixteen studies had fewer than 50 participants. Regarding the risk of bias, none of the studies could blind participants and only seven studies blinded assessors. The lack of post-intervention follow-up made it impossible to know the long-term and preventive effect of VRSs on the functional ability of older persons.

## 5. Conclusions

As a conclusion, our findings suggest that the use of VRSs is an innovative and feasible technique to support and improve the functional autonomy of community-dwelling older adults. However, clinical trials of a higher methodological quality are necessary to increase the level of knowledge of its actual effectiveness.

## Appendix A

**Table A1:** Key search terms.

Category	Key Search Terms
Population	Aging, elderly, geriatr *, gerontology, late life, older adults, oldest, seniors, old age.
Intervention: VRT	(“Wii *” OR “gaming technology” OR “active videogame” OR nintendo OR “video game*” OR exergam* OR Wii OR kinect OR Xbox OR “Play Station” OR “Xavi Sports”) OR (“virtual reality” OR virtual-reality tecnhology OR “Virtual navigation” OR “Virtual environment” OR “Simulated reality” OR “virtual rehabilitation” OR vr-based OR “virtually simulated” OR “use-computer interface”)
Outcome	Activities of daily living, daily living activities, daily self-care activities, adl, daily life activity *, functional Independence, physical functional performance, transfer, bathing, grooming, eating, feeding, functional mobility, walking, walking capacity, ambulation, gait, mobility, showering, toilet hygiene, toileting, personal hygiene, self-care, self-feeding,
Study and trial designs	clinical trial, controlled clinical trial, double blind, evidence-based, evidence synthesis, feasibility study, health technology assessment, intervention, longitudinal, multicenter study, pilot, random allocation, randomized controlled trials, standard of care, treatment outcome, validation study

## Appendix B

**Characteristics of Included Studies (*N* = 23)**											
**Author**	**Evidence Level Design**	**Participants**				**Intervention**				**Follow-ups**	**Result**
		n	Age (yrs) Mean (SD) Range	Sex	Inclusion Criteria	Placement	Experimental Group	Control Group	Outcome Measures		Results
Chao 2014 [48]	Level II	n = 32 EC = 16 CC = 16	85 (79–91)	67 % Female	>65 years; independent walking; able to read and speak English; able to follow instructions	Assisted living Buffalo (EEUU)	SAHA + Nintendo Wii Fit 4 weeks 2 times/week	Health educational session	BBS; TUG SMWT; GDS FES; SF8	No follow-up	The experimental group improved gait speed, reduced depressive symptoms and increased confidence in activities of daily living. The control group did not show improvement in any result.
Choi 2019 [58]	Level I RCT	n = 57 EC = 28 CC = 29	69–85	84% Female	>65 years; < 26 MCA; able to communicate	Welfare center, Korea	VPK Exercise 6 weeks 2 times/week	Home exercise program: flexion, curl-ups, sideways leg lifts, prone leg lifts and supine leg lifts.	BBS; FSST; TUG; OLST; MCA; ACT	No follow-up	VPK improves physical and cognitive functions. VKP exercise induces significant increases in postural control and muscular strength.
Crotty 2011 [38]	Level I RCT	n = 44 EC = 22 CC = 22	84 (80–88)	79% Female	>65 years; <150 kilos; independent walking; MEC: 21–30; good level of vision	Acute care hospital, Australia	Nintendo Wii 2 weeks 5 times/week	Strength, balance, aerobic exercises	TUG; BBS; SPPB; FIM; AIDL	4 weeks after intervention	Wii is feasible for providing treatment to elderly patients. It can improve mobility and balance in comparison with traditional approaches.
Daniel 2012 [33]	Level I RCT	n = 23 EC = 16 CC = 7	76 (55–86)	57% Female	>65 years; 1–2 of the characteristics of frailty	Senior centers and residential living centers Texas, US	Nintendo Wii Fit 15 weeks 3 times/week	Traditional senior fitness program	Senior fitness test; body weight; BES, CHAMPS; LLFDI SF-36.	No follow-up	Both groups improved scores in ABC and CHAMPS scales. The experimental group increased caloric expenditure.
Farzin 2018 [39]	Level I ECA	N = 25	55 (51–59)	68% Female	>55 years; absence of a) history of neurological impairments, b) psychiatric disorders, c) learning disabilities, d) cerebrovascular disease	Older Adult University Program Kuala Lumpur, Malaysia	Virtual Week Board Game 6 weeks 1 time/week	Usual routine activities	GAS; IADL; PRMQ; PMT	4 and 12 weeks after the intervention	VWB training improved performance and activities of daily living and reduce depression and anxiety levels.
Htut [51]	Level I RCT	n = 84 EC:VR = 21 EC:BE = 21 EC:PE = 21 CC = 21	75 (70–80)	37% female	65–85 years MEC > 23 BI = 100	Residential aged-care Yangon, Myanmar	Xbox Kinect Cognitive Stimulation Balance exercise 8 weeks 3 times/week	Without intervention	TUG; 5 TST FES; MoCA BBS	No follow-up	VR group improved both physical and cognition performance, while BE was effective in enhancing cognition. VR and BE may help fall concern in older persons.
Hughes 2014 [40]	Level II	n = 20 EC = 10 CC = 10	77.4 (72–83)	70% Female	MYHAT Cognitive Classification	Community-dwelling adults Pittsburgh, US	Nintendo Wii Sports 24 weeks 1 time/week	Without intervention	CAMCI; CRSQ AIVD GAIT: 6 m	No follow-up	The experimental group improved in overall cognition, perceived cognitive ability and gait speed.
Hung 2019 [42]	Level I RCT	n = 24 EC = 12 CC = 12	68 (66–72)	57% female	>60 years; medical diagnoses of diabetes; independently ambulatory; MEC > 24	Community-dwelling adults Taipei, China	Xavi Sport VR 6 weeks 3 times/week	Without intervention	TUG; BBS UST; MFES	6 weeks after the intervention	VR showed positive effects on functional balance. BBS, UTS and TUG test scores significantly improved after intervention.
Keogh 2014 [49]	Level II Quasi-experimental	n = 34 EC = 19 CC = 15	83 (75–91)	55% Female	>65 years; walk 10 min without aid; cognitive capacity to understand instructions	Residential aged-care Gold Coast, Australia	Nintendo Wii Sports 8 weeks	Usual routine activities	FSST RAPA WhOQOL-BREF semistructured group Interview	No follow-up	The intervention group showed a statistically significant improvement in strength in upper limbs, levels of physical and psychological activity and quality of life after participating in virtual reality programs with Wii.
Lee 2013	Level I RCT	n = 64 EC = 32 CC = 32	74 (69–79)	71% Female	>65 years; diagnosis of type 2 DM; able to communicate	Community-dwelling adults Seoul, Korea	Sony PlayStation and EyeToy 10 weeks 2 times/week	Health education on diabetes management	BBS TUG STS Gait Rite FES	No follow-up	VR can be used to prevent falls and improve the quality of walking.
Maillot 2012 [7]	Level I RCT	n = 32 EC = 16 CC = 16	71 (65–78)	84% Female	Recommendation to Canadian Health Network. Never play video games and living a sedentary lifestyle	Community-dwelling adults Paris, France	Nintendo Wii Sports 14 weeks	Without intervention	MAQ; GDS MMSE, SFT, TNT, ST, MRT, SST	No follow-up	Improvement in the VRTI group in cognitive and physical tests. There were no differences in visuospatial function.
Montero 2019 [43]	Level II Quasi-random	n = 977 EC:508 EE:469	75 (72–78)	59% female	<70 years; able to walk independently (with or without walking aids)	Community-dwelling adults Cataluña, Spain	Exergame Wii Fit with Wii Balance Board	Usual routine activities	Tinetti Test Short FES-I UST	9 weeks after intervention	Found no effect of balance training usie on balance or falls. A reduced fear of falling was found at 3 months, but no longer at 1 year. The authors think this may be a chance finding.
Morat 2019 [46]		n = 45 VOL = 15 VOL + US = 15 Cc = 15	69 (64–74)	62% female	<60 years; healthy	Community-dwelling adults Cologne, Germany	Dvidat, Senso Dvidat Senso+ Postoromed 8 weeks 3 times/week	Without intervention	TUG, Y-balance test, motor dual-task	No follow-up	Exergames under stable and unstable conditions are a feasible training tool with high adherence rates to improve functional balance and calf strength. Exergames, especially under unstable conditions, improve factors that are relevant for fall prevention, such as balance, functional mobility and strength.
Mugueta 2019 [34]	Level I RCT	n = 40 EC = 20 CC = 20	84 (77–91)	60% female	>65 years; >90 MBI; <10 SPPB; no physical exercise	Elderly day centers Bilbao, Spain	Exergame FRED, Kinect 6 weeks 3 times/week	Without intervention	BI; SPPB EuroQol	No follow-up	FRED Exergame reduced frailty risk: 14.7% less than control group No difference in EuroQol or BI.
Nicholson 2015 [50]	Level II Two groups, nonrandomized studies	n = 43 EC = 21 CC = 22	74 (69–79)	76% Female	>65 years; not involved in balance exercises for the last 3 months; independent walking; adequate visual capacity; without progressive or terminal illness, acute illness or unstable chronic illness	Community-dwelling adults. Sunshine Coast, Australia	Nintendo Wii Fit 6 weeks 3 times/week without supervision	Without intervention	TUG; FES STS; PACES; FR	No follow-up	The experimental group improved in all variables but only left lateral reach showed statistical significance.
Orsega-Smith 2012 [35]	Level I RCT	n = 30 EC = 20 CC = 9	71 (66–83)	92% Female	>63 years	Community-dwelling Older Adults	Nintendo Wii Fit: balance and yoga 4 weeks 8 weeks	Without intervention	ABC/FES; ADL BBS; TUG; CS	No follow-up	The experimental group improved transfers, balance and ADLs. The control group did not show any changes.
Portela [36]	Level I RCT	n = 65 EC = 23 CC = 42	79 (78–80)	61% Female	>65 years; good level of vision and hearing; stand for 2 min without upper limb support	Community-dwelling Older Adults	Nintendo Wii 12 weeks 1 time/week	Traditional senior fitness program	BI; BBS; MMSE SF36	No follow-up	The supervised use of Wii had an impact on physical improvement. The unsupervised use of Wii had a positive impact on vitality and mental health.
Schoene 2013 [47]	Level I RCT	n = 37 EC = 15 CC = 17	77.5 (73–82)	-	Walking independently 20 m; independent for ADLs	Independent-living units Sydney, Australia	Exergame DDR Stepmania 8 weeks 2 times/week	Usual routine activities	CSRT RT/CRST MT; 5STS; TUG TRIAL MAKING FES; PPA; AST	No follow-up	VR is safe and can be applied at home. It improves physical and cognitive parameters for fall prevention.
Singh 2013 [44]	Level I RCT	n = 38 EC = 16 CC = 16	62.5 (58–69)	100% Female	>65 years; independent walking	Senior citizens’ club Kuala Lumpur, Malaysia	Wii Balance Board 6 weeks	Traditional senior fitness balance program	OPI; TST; TUG	No follow-up	VR improves agility, balance and functional mobility. There was no significant difference between groups.
Stanmore 2019 [45]	Level I RCT	n = 106 EC = 56 CC = 50	78 (58–96)	78 % Female	>55 years; mental capacity; able to speak English; able to watch television from 2 m distance; able to use VR	Assisted living facilities Manchester and Glasgow, UK	Exergame FAME OTAGO 12 weeks	Physical exercise	BBS; FES TUG; GDS	No follow-up	VR exergame improved balance, pain and fear of falling and are a cost-effective fall prevention strategy in assisted living facilities.
Tollar 2019 [37]	Level I RCT	n = 83 EC-CYC = 27 EC-EXE = 28 CC = 28	69.6 (66–72)	53% Female	>60 years; >24 MMSE; >40 BDI; mobility difficulty	Hospital Somogy, Hungary	Xbox or bicycle ergometer 5 weeks 5 times/week	Usual routine activities	6MWT; SE-ADL; DGI; MAP; SF-36; WOMAC	No follow-up	Xbox and cycling improved general health-related QoL and walking capacity. Xbox improved the SE-ADL (7.3%)
Gomes 2019 [41]	Level I RCT	n = 30 EC = 15 CC = 15	84 (78–90)	93% female	>60 years; able to walk independently; normal or corrected visual acuity; good hearing; no previous experience with VR	Sao Paulo Brazil Hospital	Nintendo Wii Fit 7 weeks 2 times/week	Advice regarding the importance of physical activity	FGA; MoCA; FES	4 weeks after intervention	VR Wii-Fit was feasible, acceptable and safe for frail older adults and improved their postural control and gait. There were no effects on cognition, mood or fear of falling.
Liao 2019 [52]	Level I ECA	n = 52 EC = 27 Cc = 25	81 (73–89)		>65 and <95 years; the presence of at least one of the 5 frailty characteristics defined by Fried	Daycare centers Taiwan	Kinect: Tano, LongGood 12 weeks 3 times/week	Combined exercise: Resistance, aerobic and balance	IPAQ: IMC; TUG; grip strength; FES	No follow-up	Kinect-based exergaming is at least as beneficial as combined exercise in the prefrail and frail elderly.
ABC: Activity-specific Balance Confidence; ACT: Arm Curl Test; ADL: Activities of Daily Living; AIDL: Activity Instrumental Daily Living; AST: Alternate Step Test; BBS: Balance Berg Scale; BE: Brain Exercise; BI: Barthel Index; BDI: Beck Depression Inventory; CAMCI: Computer Assessment of Mild Cognitive Impairment; CHAMPS: Community Healthy Activities Model Program for Seniors questionnaire CSRQ: Cognitive Self-Report Questionnaire; CRST-RT: Choice Stepping Reaction Time; CS: Chair Stand; FES: Falls Efficacy Scale; DGI: Dynamic Gait Index: FGA: Functional Gait Assessment; FIM: Functional Independence Measure; FPA: Foot Placement Accuracy; FR: Functional Reach; FSST: Four Square Step Test; GAS: Geriatric Anxiety Scale; GDS: Geriatric Depression Scale; IADL: Instrumental Activities of Daily Living scale; Level I: randomized controlled trials; Level II: two groups, nonrandomized studies (e.g., cohort, case–control); MAQ: Modifiable Activity Questionnaire; MBI: Modified Barthel Index; MCA: Montreal Cognitive Assessment; MEC: Mini-Examen Cognoscitivo; DM: Diabetes Mellitus; MAP: Mean Arterial Pressure; MCA: Montreal Cognitive Assessment; MFES: Modified Falls Efficacy Scale; MMSE = Mini Mental State Examination; MoCA: Montreal Cognitive Assessment; MRT: Matrix Reasoning Test; MT: Movement Time; OLST: One-Leg Stance Test; OPI: Overall Performance Index; PPA: Physiological Profile Assessment score; PACES: Physical Activity Enjoyment Scale; PE: Physical Exercise; PMT: Prospective Memory Tasks; PRMQ: Prospective Retrospective Memory Questionnaire; RAPA: Rapid Assessment Physical Activity scale; RCT: Randomized Clinical Trial; SFT: Senior Fitness Test; SF8: Short Form 8; SF36: Short Form 36; ST: Stroop Test; SE-ADL: Schwab–England Activities of Daily Living scale; SF-36: Short Form 36 Health Survey; SMWT: Six-Minute Walk Test; SPPB: Shot Physical Performance Battery; SST: Spatial Span Test TMT: Trail Making Test; TST: Ten Step Test; TUG: Timed Up and Go; UST: Unipedal Stance Test; VR: Virtual Reality; WHOQL-BREF: World Health Organization Quality of Life; WOMAC: McMaster Universities Osteoarthritis Index; VPK: Virtual Kayak Paddling; 5STS = Five Times Sit-to-Stand; 6MWT: 6-min Walk Test.											

## Appendix C

**Table A2:** Virtual Reality Types, Video Game Consoles and Exergames of Included Studies.

Author	Virtual Reality Type	Videogame Console and Technical Assistance	Exergame
Chao, 2014 [48]	Active Video Game	Nintendo Wii + Balance Board Walker was placed around the balance board	Wii Fit: Jogging; LungE; Penguin slide Table tilt; Chair; Deep breathing
Choi, 2019 [58]	Specific Virtual Reality	Virtual kayak paddling + a projected video	Kayaking
Crotty, 2011 [38]	Active Video Game	Nintendo Wii + Balance Board Chair around the balance board	Wii Fit
Daniel, 2012 [33]	Active Video Game	Nintendo Wii + weighty vest	Wii Sport, tennis, boxing, boling
Farzin, 2019 [39]	Specific Virtual Reality	Virtual Week (VW) Board Game	VW includes 8 to 10 PM tasks (four regular, four irregular and two stop clock tasks) which allow for exercising almost all types of everyday PM tasks (e.g., self-care, medication adherence, keeping social appointments)
Htut, 2018 [51]	Active Video Game	X-Box 360 + Kinect	Light Raise, Virtual Smash Stack ’em Up, One Ball Roll, Pin Push, Super Saber, Target Kick, Play Paddle Panic, Body Bally, Bamp Bash
Hughes, 2014 [40]	Active Video Game	Nintendo Wii	Wii Sports: bowling, golf, tennis, and baseball Wii Play: Wii Resort Boom Blox
Hung, 2019 [42]	Active Video Game	Xavi Sport + stepping mat	Set-up, stepping exercise, hamster game, drumming game
Keogh, 2014 [49]	Active Video Game	Nintendo Wii	Wii Sports: bowling, golf, tennis, baseball, boxing
Lee, 2013 [59]	Active Video Game	Play Station + EyeToy	Wishi Washi: window washing, Keep Ups, Bowling, Bubble Pop, Boot Camp, Kung Foo
Maillot, 2012 [7]	Active Video Game	Nintendo Wii	Wii Fit Wii Sport: tennis
Montero, 2019 [43]	Active Video Game	Nintendo Wii	Not specified
Morat, 2019 [46]	Specific Virtual Reality	Dividat Senso Swing and Posturomed	Targets, Divided, Simon, Flexi, Snake, Tetris, Habitats, Birds, Hexagon
Mugueta, 2019 [34]	Specific Virtual Reality	FRED + Kinect	FRED entails several scenarios, with each one representing one or more steps in a simplified process to enable txakoli to be produced
Nicholson, 2015 [50]	Active Video Game	Nintendo Wii + Balance Board	Soccer heading, penguin slide, ski slalom, ski jump, table tilt, snowball fight, perfect 10, and tightrope walking
Orsega Smith, 2012 [35]	Active Video Game	Nintendo Wii + Balance Board	Wii Fit: Balance Games: Penguin Slide,’ ‘Table Tilt,’ ‘Ski Slalom,’ ‘Balance Bubble’, Hula Hood, Snowboard Slalom; Wii Fit: Yoga: deep breathing, half-moon, palm tree
Portela [36]	Active Video Game	Nintendo Wii	Wii Sport
Schoene, 2013 [47]	Active Video Game: Stepmania	Television + step pad + computer unit	Open-source modified DDR game Stepmania: pressure sensitive panels which represent stepping direction and additional cognitive load (“bomb” for dodge)
Singh, 2013 [44]	Active Video Game	Nintendo Wii+ Balance Board	Ski Slalom, Table Tilt, Penguin Slide, Soccer Heading, Tight Rope Walk, Perfect 10, Tilt City
Stanmore, 2019 [45]	Specific Virtual Reality	MIRA Exergames + Kinect	Side Taps: Atlantis, Catch, Izzy the Bee, Move, Follow; Jugger, Firefly, Catch, Follow, Move; Sit to Stand: Powerhouse Bid, Atlantis, Izzy the Bee; Squats: Izzy the Bee, Atlantis, Catch, Move, Follow; Hip Frontal Flexion: Animals, Atlantis, Airplane, Catch, Follow, Colour Clouds, Move, Piano; Elbow Flexion: Grab, Catch, Follow, Atlantis, Izzy the Bee, Move, Firefly, Piano, Jugger; Full Body Turn: Animals, Catch, Firefly, Colour Clouds, Follow, Jugger, Basketball, Move; General–Shoulder: Atlantis, Catch, Firefly, Follow, Izzy the Bee, Jugger, Move; Hip Abduction: Atlantis, Catch, Izzy the Bee, Move, Follow, Fireflies, Jugger, Seasons; Knee Flexion: Grab, Catch, Follow, Atlantis, Izzy the Bee, Move, Firefly, Jugger; Shoulder Abduction: Atlantis, Follow, Catch, Izzy the Bee, Move, Firefly, Jugger, Follow, Memory scape
Tollar, 2019 [37]	Specific Virtual Reality	Exer Program designed to improve postural control, gait mobility, gait stability, turning and balance + Kinect	Space Pop trains spatial orientation through target reaching with arms, legs and whole body and Just Dance prompts users to generate and combine movement sequences
Gomes, 2018 [41]	Active Video Game	Nintendo + Balance Board	Wii Fit Plus: Table Tilt, Rhythm, Parade, Obstacle Course, Single Leg Extension, Tilt City, Basic Step, Penguin Heading Soccer, Basic Run, Torso Twist
Liao, 2019 [52]	Active Video Game	Tano and LongGood software packages + Kinect	Tai-chi exercise, resistance and aerobic exercises, balance game

## Appendix D

**Table A3:** Grading of Recommendations Assessment, Development and Evaluation (GRADE): Evidence Profile: SRV vs. control on functional autonomy: ADL.

Evidence Profile: SRV vs. Control on Functional Capacity: ADL			Evidence Profile: SRV vs. Control on Functional Mobility		
N° of studies: 8 (10 comparisons) Type of studies: RCT Intervention: Virtual Reality Systems on ADL Control: TPE, ADL, programs of health education or non-intervention N° of patients: 212			N° of studies: 22 (38 comparisons) Type of studies: RCT Intervention: Virtual Reality Systems Control: physical exercise, programs of health education or non-intervention N° of patients: 1097		
GRADE Factor	Importance	Explanations	GRADE Factor	Importance	Explanations
Limitations in study design or execution (risk of bias)	Moderate ↓1 level	Downgrading for risk of bias was not considered because most information is from studies at moderate risk of bias Moderate risk of bias Rate down one level	Limitations in study design or execution (risk of bias)	Moderate ↓1 level	Downgrading for risk of bias was not considered because most information is from studies at moderate risk of bias. Moderate risk of bias Rate down one level
**Inconsistency results**	Serious ↓1 level	Downgrading for inconsistency was considered because I^2^ = 79%.	**Inconsistency results**	No serious inconsistency	Downgrading for inconsistency was not considered because I^2^ = 58%
**Indirectness evidence**	No serious indirectness	In none of the studies were there substantial differences between the population, intervention or outcomes measured in the studies and those established in the systematic review.	**Indirectness evidence**	No serious indirectness	In none of the studies were there substantial differences between the population, intervention or outcomes measured in the studies and those established in the systematic review.
**Imprecision**	Moderated ↓1 level	The OIS was calculated, the resulting OIS being 276 participants with MDS = 0.3. The number of patients included in the meta-analysis is 212, moderately lower to the OIS. Downgrading for imprecision was considered.	**Imprecision**	No serious imprecision	The OIS was calculated, the resulting OIS being 276 participants with MDS = 0.3. The number of patients included in the meta-analysis is 1097, far superior to OIS. Downgrading for imprecision was not considered.
**Publication bias**	Not serious	Downgrading for publication bias was not considered because the DOI plot shows no asymmetry with LFK = 0.36.	**Publication bias**	Serious ↓1 level	Downgrading for publication bias was considered because the DOI plot shows marked asymmetry with LFK = 3.3 (major asymmetry).
**Magnitude of effect**	Moderated ↓1 level	SMD = │0.31 │ Upgrading for large magnitude of effect was not considered.	**Magnitude of effect**	Not serious	SMD =│0.58│ Upgrading for large magnitude of effect was not considered.
**Dose-response gradient**		Not considered.	**Dose–response gradient**		Not considered.
**No plausible confounders**		Not considered because studies in this meta-analysis are not observational.	**No plausible confounders**		Not considered because studies in this meta-analysis are not observational.
**Quality rating**	**Very low quality:** we are moderately confident in the effect estimate; the true effect is likely to be close to the estimate of the effect, but there is a possibility that it is substantially different (GRADE Working Group grades of evidence)		**Quality rating**	**Moderate–low quality****:** we are moderately confident in the effect estimate; the true effect is likely to be close to the estimate of the effect, but there is a possibility that it is substantially different (GRADE Working Group grades of evidence)	

**Abbreviations: ADL:** Activities of Daily Living; **LFK:** LFK Index; **MCID:** Minimum Clinically Important Difference; **RCT:** Randomized Controlled Trial; **OIS:** Optimal Information Size. **VRS:** Virtual Reality System.
